# Association between Knowledge of Zika Transmission and Preventative Measures among Latinas of Childbearing Age in Farm-Working Communities in South Florida

**DOI:** 10.3390/ijerph16071257

**Published:** 2019-04-09

**Authors:** Naiya Patel, Moneba Anees, Reema Kola, Juan Acuña, Pura Rodriguez de la Vega, Grettel Castro, Juan G. Ruiz, Patria Rojas

**Affiliations:** 1 Herbert Wertheim College of Medicine, Florida International University, Miami, FL 33199, USA; npate086@med.fiu.edu (N.P.); manee002@med.fiu.edu (M.A.); rkola004@med.fiu.edu (R.K.); rodrigup@fiu.edu (P.R.d.l.V.); gcastro@fiu.edu (G.C.); jruizpel@fiu.edu (J.G.R.); 2 Robert Stempel College of Public Health & Social Work, Florida International University, Miami, FL 33199, USA

**Keywords:** Latinas, Zika, knowledge, transmission, prevention

## Abstract

Zika infection, an otherwise usually mild disease, is of serious public health concern due to the potential teratogenic effects of the virus. The incidence of Zika infection is difficult to document since it is mostly asymptomatic and detection of those carrying Zika is usually not possible. Currently, there is no vaccine for Zika; therefore, use of personal preventative measures is the only method of avoiding transmission. The aim of this study was to evaluate the association between knowledge of Zika transmission and the use of preventive measures among Latinas of childbearing age who lived in or near farm-working communities in South Florida. A secondary data analysis was performed on a cross-sectional study, sampling 100 Latina women aged 18–50 years. Sixty-nine percent demonstrated a high degree of knowledge of Zika transmission, and 68% were categorized as taking good preventative measures. Women with high knowledge were 5.86 times more likely to take good preventative measures than those with no knowledge (*p*-value = 0.05). Knowledge was associated with more preventative measures. Therefore, it is essential to further investigate this relationship in order to develop effective public health interventions for this population.

## 1. Introduction

Zika virus is an arbovirus belonging to the *Flavivirus* genus and the *Flaviviridae* family [1]. Similar to dengue and yellow fever, Zika infection is mainly transmitted by *Aedes aegypti*. Besides vector-borne via mosquitos, other routes of transmission include sexual, maternal–fetal, and transfusion-associated transmissions. The first cases of human infection were reported in Africa, then the disease spread to Southeast Asia [2]. In 2007, there was an epidemic in the islands of Yap in Micronesia [2]. Infection has since widely spread throughout the Americas. In February of 2016, the World Health Organization (WHO) declared Zika a public health emergency of international concern due to the link between Zika and neurological birth defects [3,4]. A Zika infection during the gestational period of pregnancy is associated with central nervous system abnormalities such as microcephaly among newborns and other birth defects such as hearing loss and abnormal eye development. Within the last three years, out of 6848 possible Zika-associated pregnancies in the United States, there were 283 cases of live-born infants with Zika-associated birth defects and 17 miscarriages [5]. Zika virus also presents a financial burden for nations. The potential nationwide economic burden is estimated to range from $183.4 million to $1.2 billion for direct medical costs and productivity losses [6].

Zika is currently active in the United States, and new cases have been reported to the Center for Disease Control in 2018 [7]. Among the general population, 78% of cases are asymptomatic, but among pregnant women, 55% are asymptomatic [8]. The proportion varies greatly among different subgroups of the population. The majority of cases of Zika virus infection are asymptomatic, making it difficult to diagnose. Due to the lack of vaccines or treatment, public health officials in the United States have emphasized non-pharmaceutical interventions for prevention [9]. Environmental strategies for vector control focus on elimination of breeding sites such as standing water and indoor spraying. Behavioral strategies include use of insect repellant, minimal skin exposure, use of condoms, and limiting travel to endemic regions. Clinical strategies include availability of Zika reproductive health termination services, screening for blood supply, and access to testing. These public health efforts will reduce mosquito density, risk of transmission, and the likelihood of birth defects [9]. Zika virus presents a serious issue in the United States, especially in South Florida due to its geographical location and climate. In 2016, Florida had a total of 1107 cases, the largest number of confirmed cases of Zika virus compared to other states [7]. South Florida has more risk factors for the spread of Zika because it has a subtropical climate and is a central hub for those traveling to and from areas with active Zika virus transmission [10]. Clinical and financial implications contribute to the relevancy of Zika in South Florida.

After the 2016 outbreak in South Florida, knowledge of Zika virus has been prevalent in the United States [11]. However, there is limited research on the association between knowledge and preventative practices against Zika. Borges et al. found that implementation of behavioral action was significantly associated with knowledge of Zika transmission among women of childbearing age [12]. Although general awareness of Zika as a congenital syndrome was found to be high at 98.6%, knowledge about sexual transmission was only 50.2%. The team found that there was a higher use of condoms among those who had knowledge of sexual transmission (*p* = 0.006). Borges et al. found that knowledge of sexual transmission is significantly associated with an increased usage of condoms as a means of protection from the virus. This supports the concept that knowledge of transmission is associated with preventative measures. Therefore, we hypothesize that knowledge of other routes of Zika transmission will serve as a basis to initiate the associated preventative measures. For example, knowing that Zika is transmitted via mosquitos will lead to less skin exposure, use of mosquito repellant, and installation of window screens to reduce the likelihood of mosquito bites. Without knowing its mechanism of transmission, individuals cannot deduce preventative interventions to block these routes of transmission. This study aims to examine the association between knowledge of Zika transmission and use of preventive measures among Latinas living in farm-working communities in South Florida. This population, which is important for this area with high prevalence of Zika, has not been previously studied. 

## 2. Materials and Methods

### 2.1. Participants

The study used a convenient non-clinical community sample. One hundred Latina women were recruited and interviewed. Inclusion criteria were as follows: living in a farm-working community in South Florida, being 18–50 years of age, speaking and understanding Spanish, willingness to be interviewed for 90 min, and having participated in a previous non-clinical intervention study. Participants who refused to answer any of the following questions and participants who had not previously heard about Zika were excluded. All subjects gave their informed consent for inclusion before they participated in the study. The study was conducted in accordance with the Declaration of Helsinki and the protocol was approved on 18 April 2017 by the ethics committee of IRB-17-0116-AM02. The questionnaire was designed to explore the knowledge, attitudes, and behaviors concerning Zika virus. Data was obtained from a semi-structured 90-min face-to-face questionnaire conducted in Spanish. The questionnaire was adapted from the World Health Organization resource pack titled “Knowledge, Attitudes and Practice surveys Zika virus disease and potential complications” [13].

Interviews were conducted by a community leader trained by a PhD level and a master level social worker. Eligible women were called or approached in their neighborhoods and asked to participate in the study. Over 95% of the women approached agreed to participate. Study data were collected and managed using Research Electronic Data Capture (REDCap), a software toolset and workflow methodology for electronic collection and management of research and clinical trial data developed by Vanderbilt University and a consortium of more than 1000 partners.

### 2.2. Study Variables

Knowledge of Zika virus transmission was assessed by number of correct methods of transmission reported. The responses encompassed 15 alternative modes of transmission (4 correct, 11 incorrect), 1 item for “other” modes of transmission (always incorrect), 1 for “I don’t know”, and 1 for “I prefer not to answer” (proxy for “I don’t know”). One of the items (transmitted via virus) was ambiguous and was eliminated. There were four correct mechanisms enumerated in the following items: from mosquito bite, sexual relations, blood transfusion, and mother to fetus. The other responses demonstrated incorrect transmission mechanisms, and the correct answer should be no. Each correct answer was scored 1 point and each participant was assigned a summative score with a maximum score of 14. A score of 11 or greater was categorized as high knowledge. A score of 10 or less was categorized as low knowledge. A score of zero was classified as no knowledge. The participants were divided into three categories: those with no knowledge of Zika transmission, low knowledge of Zika transmission, and high knowledge of Zika transmission.

Level of preventative measures was assessed by the type of measures taken to prevent contraction of Zika. The responses included a total of 16 possible answer choices. Out of 16 responses, the following eight were appropriate and feasible: using mosquito repellant, wearing clothing to cover skin, fumigating the living areas, placing mosquito screens on windows and doors, using condoms, cleaning containers with standing water, removing standing water, and using mosquito nets at night. Good level of preventative measures taken was defined as the use of at least one appropriate measure. 

Age was stratified into three categories: 18–24, 25–34, and 35–50 years. Annual household income was divided into less than $25,000 and greater than $25,000 (USD). Employment status was determined by employed or not. Education was stratified into three categories: no/some high school, completed high school, and above high school. No/some high school was defined as not having attended or not having finished high school. Completed high school was defined as having obtained a high school diploma. Above high school was defined as having attended a university, obtaining a bachelor’s degree or obtaining a master’s degree. English proficiency was divided into three categories: none, some, and high. Some English proficiency was defined as a response of understanding and speaking English a little. High English proficiency was defined as a response of understanding and speaking English well. Source of information received about Zika was sorted into three categories: media, community, and health care. Media sources include radio, television, posters, newspapers, internet, websites, text messages, and smartphone applications. Community sources include family members, friends, community meetings, local healer, traditional birth assistant, church, campaign, announcement from government, international organization, and local or national organization. Health care sources include health care workers in a health center or community, private doctor, or pharmacy. First time heard about Zika was divided into more than a year ago and less than a year ago. Location of birth was sorted into those born in the United States and outside of the United States. Perception of risk was assessed by a summation score of four risk questions: (1) Is it possible to contract Zika in your community? (2) Is Zika virus associated with microcephaly? (3) Do you know anyone in your community that contracted Zika virus? (4) Do you think Zika is an important problem in your community? Responding yes to three or more questions was classified as high risk.

### 2.3. Statistical Analysis

Data was analyzed using IBM Statistical Package for the Social Sciences (SPSS) Version 23 software (IBM Corp., Armonk, NY, USA). Univariate analysis was used to assess for variable quality and frequency distributions. Descriptive statistical measures were computed and presented for each variable. Chi-squared test was used to analyze the bivariate associations between potential confounders and the exposure as well as the association between potential confounders and the outcome. Percent distributions for exposure and outcome in relation to potential confounders was calculated. The correlation matrix between predictors was examined to check for collinearity. Multivariable logistic regression was conducted to calculate the independent effect of the knowledge of Zika and the use of preventative measures. Odds ratios and 95% confidence intervals were calculated. In the multivariable analysis, confounders were included if they had a statistically significant association with the exposure and outcome (*p*-value equal to or less than 0.20) or if they changed the odds ratio of the exposure by at least 15%.

## 3. Results

Among the one hundred women that completed the survey, three women reported that they had never heard of Zika and, therefore, were excluded from the study, leaving a final sample of 97 women. Of the remaining 97 women, 82.5% were from a farm-working community, while 17.5% were from the nearby area. As seen in Table 1, the majority of women surveyed were between the ages of 35 and 50 (*n* = 53, 55%), had an income less than $25,000 (*n* = 66, 68%), and had some English proficiency (*n* = 48, 49%). It was found that 7% of participants had no knowledge of Zika transmission, 24% had low knowledge of Zika transmission, and 69% had a high knowledge of Zika transmission. There is an itemized analysis of the knowledge of Zika transmission in Appendix A Table A1. In total, 66 participants (68%) were classified as taking good preventative measures, 73% of whom had high knowledge of transmission.

Baseline characteristics were evenly distributed among participants with different levels of knowledge about Zika transmission, except for income (Table 1). The highest proportion of annual income greater than $25,000 was observed among women with high knowledge about Zika transmission (41.8%, *p* = 0.01). The percentage of women with an income of lower than $25,000 was highest among women with low knowledge of Zika transmission (91.3%, *p* = 0.01). 

As indicated in Table 2, there were no significant differences in the distribution of levels of preventative measures according to any of the measured variables except for previous knowledge of Zika transmission. The percentage of women who undertook poor levels of preventative measures was significantly higher among women with no knowledge of Zika transmission (71%, *p* = 0.03). The percentage of women who took a good level of preventative measures was significantly higher among women with high knowledge of Zika transmission (73%, *p* = 0.03). There is an itemized analysis of the preventative measures in Appendix A Table A2. 

Table 3 shows the unadjusted and adjusted odds ratios, and their respective 95% confidence intervals, for the association between knowledge of Zika and preventative measures. Prior to adjustment, we found a statistically and clinically significant association between knowledge of Zika and use of preventative measures. The odds of use of good level of preventative measures increased with increased knowledge of Zika transmission but it was only significant for those women with high knowledge (OR = 6.81, 95% CI: 1.21–38.25). After adjusting for income levels and English proficiency, the magnitude of the association decreased but remained statistically significant (OR = 5.86, 95%CI: 1.01–34.10). 

## 4. Discussion

Our results support our hypothesis that knowledge of Zika transmission is significantly associated with level of preventative measures taken. Women with either low or high knowledge are approximately six times more likely to take good preventative measures than those without knowledge. This result supports the current literature on Zika prevention and provides insight for future studies to suggest public health interventions based on our results. 

Current literature, such as the study conducted by Curry et al., has explored the knowledge and perceptions of Zika virus among reproductive-aged women living in United States and found participants to have a high level of knowledge of Zika virus [11]. However, we found a gap in knowledge among the Latina population, as 23.7% of women had low knowledge and, notably, 7.2% of women reported no knowledge of Zika transmission. Few studies have examined the association between the degree of knowledge of Zika virus and levels of preventative measures taken among women. Moise et al. found a statistically significant association between Zika virus knowledge and the taking of preventative action (aOR 2.39, *p* = 0.01), but no significant association was found among women (aOR 2.30, *p* = 0.10) when controlling for gender [14]. Borges et al. also found that implementation of condoms as a source of protection against Zika was significantly associated with knowledge of sexual transmission of Zika among women of childbearing age [12]. Our study supports Borges et al.’s findings that there is an association between knowledge and use of preventative practices; however, it was done with a small community-based sample of childbearing-age Latina women living in farm-working communities and nearby areas. Women with high knowledge were approximately six times more likely to report good preventative measures than individuals with no knowledge of transmission (aOR 5.86, *p* = 0.05). The association found, combined with the fact that 7% reported that they had no knowledge of Zika transmission, highlights the need to provide educational interventions to ensure that this underserved Latina population of childbearing age receives accurate information regarding Zika prevention. A public health effort to increase the level of knowledge among small farm-working communities in South Florida is essential for the adoption of appropriate preventative behaviors. Efforts aimed at educating this population on the transmission modes of Zika through classes, brochures, and health fairs will increase the use of preventive measures among this group of Latinas.

Change in health behavior is influenced by demographics such as age, education, income, source of information, and perception of risk [9]. Variations in these factors present a challenge for public health efforts. Some studies have found that perceived risk influences changes in health behavior. The greater the perceived personal risk, the more likely individuals are to take preventative measures [9]. Winneg et al. compared the Zika preventative actions taken by respondents in Florida who perceived themselves to be at risk versus those who did not perceive themselves to be at risk [15]. Of the Floridians who perceived themselves to be at risk of Zika infection, 57.5% took preventative actions. Out of those who did not see themselves to be at risk of Zika infection, only 37.6% undertook some form of prevention. Our results, on the other hand, did not find that perception of risk index was significantly associated with knowledge or level of preventative measures taken; however, the questions that assessed perception of risk had a 53.6% nonresponse rate, higher than all of the other questions. This low response rate may have contributed to the lack of association found.

Variations in knowledge and level of preventative measures taken was observed across income levels and English proficiency. Differences across income levels may have occurred due to varied abilities to access education and purchase resources for preventative measures. Additionally, there were differences in the level of preventative measures taken by participants of varying English proficiency levels. This may be due to limited access to educational programming in Spanish. 

Addressing knowledge gaps in this population is crucial for prevention. It was found that media was the most frequent source of information for all participants. Despite the fact that all participants received information from the same source, they varied in degrees of knowledge. This is consistent with the findings of McDonald et al., who reported that television/radio was the most common source; however, participants rated television/radio as the least helpful sources [16]. Using the most helpful source of information could help direct public health interventions to utilize the most effective mode of communication.

There are several limitations typical of a small cross-sectional, community-based, non-clinical study. The results from this study indicate that women in farm-working communities engage in preventative care for Zika. It was, however, not assessed whether they were engaging in preventative care solely to prevent Zika virus or had already been doing so for other reasons, such as dengue and chikungunya. The cutoff value used to define degree of knowledge and level of preventative measures was guided by published literature. Furthermore, participants were asked to recall information, which could have resulted in recall bias. Additionally, participants were not randomly selected; rather, a snowball sampling approach was used during the parent study. The snowball sampling method reduces the likelihood that the sample will be a good representation of the population of interest. Lastly, of the participants who took a good level of preventative measures, we did not assess how often they were using these measures. If participants did not use preventative measures regularly, we run the risk of misclassifying those who took a poor level of preventive measures as having taken a good level of preventative measures.

Future studies can investigate different and larger sample populations to further assess the relationship between knowledge and behavioral changes. In order to assess temporality, a prospective comparison study or a randomized control trial can be conducted to see if an intervention that provides knowledge of Zika transmission directly translates to an improved level of preventative measures taken. Our study suggests that the major source of information for this population is the media and other members in the community; therefore, future interventions need to include these resources in order to reach Latina women and their families. Additionally, the low educational attainment among participants and the fact that less than one-third of the community reports high English proficiency highlights the need for low academic level Spanish language media resources as well as community-based interventions.

## 5. Conclusions

In summary, Latina women of childbearing age in South Florida are susceptible to Zika virus infection due to the lack of knowledge of the multiple routes of transmission and its associated preventative measures. The lack of an effective vaccine emphasizes the importance of preventative strategies. Our study found a positive association between the degree of knowledge of Zika transmission and the level of preventative measures taken by Latinas. Therefore, public health interventions aimed to prevent Zika virus should expand their initiatives to address the low levels of knowledge in order to increase the levels of preventative measures taken by individuals, thereby preventing transmission in South Florida. 

## Figures and Tables

**Table 1:** Sociodemographic characteristics by degree of knowledge about Zika transmission among Latinas of childbearing age in South Florida (*n* = 97).

Characteristics	No Knowledge		Low Knowledge		High Knowledge		*p*-Value
	*n* (7)	7%	*n* (23)	24%	*n* (67)	69%	
Age (years)							0.44 ^1^
18–24	1	14.3	5	21.7	9	13.4	
25–34	1	14.3	3	13	25	37.3	
35–50	5	71.4	15	65.2	33	49.3	
Annual Household Income (USD ^2^)							0.01 ^1^
<$25,000	6	85.7	21	91.3	39	58.2	
>$25,000	1	14.3	2	8.7	28	41.8	
Employment Status							0.77 ^1^
Not Employed	2	28.6	10	43.5	27	40.3	
Employed	5	71.4	13	56.5	40	59.8	
Education							0.48 ^1^
No/Some HS ^3^	4	57.1	12	52.2	36	53.7	
Completed HS	2	22.2	10	43.5	17	25.4	
Above HS	1	14.3	1	4.3	14	20.9	
English Proficiency							0.66 ^1^
None	1	14.3	9	39.1	12	17.9	
Some	3	42.9	9	39.1	36	53.7	
High	3	42.9	5	21.7	19	28.4	
Source of Information							
Media	6	85.7	20	87	59	88.1	0.83 ^1^
Community	4	57.1	15	65.2	32	47.8	0.25 ^1^
Health Care	2	28.6	12	52.2	27	40.3	0.91 ^1^
First Time Heard about Zika							0.33 ^1^
More than a year	7	100	21	91.3	59	88.1	
Less than a year	0	0	2	8.7	8	11.8	
Location of Birth							0.11 ^1^
United States	3	42.9	6	26.1	12	17.9	
Outside of U.S.	4	57.1	17	73.9	55	82.1	
Perception of Risk Index							0.69 ^1^
Low	1	14.3	3	13	5	7.5	
High	2	28.6	5	21.7	29	43.4	
No information	4	57.1	15	65.2	33	49.3	

^1^ Significance for Fisher’s exact test reported because more than 20% of the cells had expected counts of less than 5. ^2^ USD: US dollars. ^3^ HS: high school.

**Table 2:** Level of use of preventative measures by degree of Zika knowledge and sociodemographic characteristics in Latinas of childbearing age in South Florida (*n* = 97).

Characteristics	Poor Level		Good Level		*p*-Value
	*n*	%	*n*	%	
Degree of Knowledge					0.03 ^1^
None	5	71.4	2	28.6	
Low	8	34.8	15	65.2	
High	18	26.9	49	73.1	
Age (years)					0.65
18–24	6	40	9	60	
25–34	10	34.5	19	65.5	
35–50	15	28.3	39	71.7	
Annual Household Income (USD ^2^)					0.18
<$25,000	24	36.4	42	63.6	
>$25,000	7	22.6	24	77.4	
Employment Status					0.52
Unemployed	11	28.2	28	71.8	
Employed	20	34.5	38	65.5	
Education					0.55
No/Some HS ^3^	18	34.6	34	65.4	
Completed HS	7	24.1	22	75.9	
Above HS	6	37.5	10	62.5	
English Proficiency					0.14
None	10	45.5	12	54.5	
Some	11	22.9	37	77.1	
High	10	37	17	63	
Source of Information					
Media	25	29.4	60	70.6	0.19 ^1^
Community	13	25.5	38	74.5	0.15
Health Care	9	22	32	78	0.07
First Time Heard about Zika					1.00 ^1^
More than a year	28	32.2	59	67.8	
Less than a year	3	30	7	70	
Location of Birth					0.88
United States	7	33.3	14	66.7	
Outside of U.S.	24	31.6	52	68.4	
Perception of Risk Index					0.27
Low	3	33.3	6	66.7	
High	8	22.2	28	77.8	
No information	20	38.5	32	61.5	

^1^ Significance for Fisher’s exact test reported because more than 20% of the cells had expected counts of less than 5. ^2^ USD: US dollars. ^3^ HS: high school.

**Table 3:** Unadjusted and adjusted association between Zika knowledge and level of use of preventative measures among Latinas of childbearing age in South Florida (*n* = 97).

Characteristics	Unadjusted	*p*-Value	Adjusted	*p*-Value
	OR ^1^ (95% CI)		OR (95% CI)	
Degree of Knowledge				
None	Reference			
Low	4.69 (0.74–29.83)	0.10	6.10 (0.91–41.08)	0.06
High	6.81 (1.21–38.25)	0.03	5.86 (1.01–34.10)	0.05
Age (years)				
18–24	Reference			
25–34	1.27 (0.35–4,58)	0.72		
35–50	1.69 (0.51–5.57)	0.39		
Annual Household Income (USD ^2^)				
<$25,000	0.51 (0.19–1.36)	0.18	0.50 (0.17–1.48)	0.21
>$25,000	Reference			
Employment Status				
Unemployed	1.34 (0.55–3.24)	0.52		
Employed	Reference			
Education				
No/Some HS ^3^	Reference			
Completed HS	1.67 (0.60–4.64)	0.33		
Above HS	0.89 (0.28–2.82)	0.83		
English Proficiency				
None	0.71 (0.22–2.22)	0.55	0.55 (0.16–1.90)	0.35
Some	1.98 (0.71–5.55)	0.20	1.84 (0.62–5.42)	0.27
High	Reference			
Source of Information				
Media	2.30 (0.92–5.74)	0.07		
Community	1.88 (0.79–4.46)	0.15		
Health Care	2.40 (0.71–8.16)	0.16		
First Time Heard about Zika				
More than a year	1.11 (0.27-4.61)	0.89		
Less than a year	Reference			
Location of Birth				
United States	Reference			
Outside of U.S.	1.08 (0.39–3.03)	0.88		
Perception of Risk Index				
Low	Reference			
High	1.75 (0.36–8.61)	0.49		
No information	0.80 (1.80–3.57)	0.77		

^1^ OR: odds ratio; ^2^ USD: US dollars; ^3^ HS: high school.

## Appendix A

Study data were collected and managed using Research Electronic Data Capture (REDCap) electronic data capture tools hosted at Florida International University [17]. REDCap is a secure, web-based application designed to support data capture for research studies, providing (1) an intuitive interface for validated data entry; (2) audit trails for tracking data manipulation and export procedures; (3) automated export procedures for seamless data downloads to common statistical packages; and (4) procedures for importing data from external sources. REDCap is a scalable, secure, enterprise-level application. Planning, configuration, and end-user support for REDCap can be found at https://cateredcap.fiu.edu/.

**Table A1:** Itemized analysis of knowledge about Zika transmission among Latinas of childbearing age in South Florida 2017–2018 (*n* = 97).

Route of Transmission	*n*	%
Mosquito Bite	89	91.8
Drinking Contaminated Water	13	13.4
Bathing in Contaminated Water	13	13.4
Sexual Intercourse	28	28.9
Coughing and Sneezing	4	4.1
Breast Milk	5	5.2
Vaccines	3	3.1
Fumigation	2	2.1
Larvicides	0	0
Pesticides	3	3.1
Blood Transfusion	8	8.2
Dirty Environment	8	8.2
Mother to Child	13	13.4

**Table A2:** Itemized analysis of preventative measures for Zika transmission among Latinas of childbearing age in South Florida 2017–2018 (*n* = 97).

Preventative Measure Taken	*n*	%
Use of Mosquito Nets at Night	10	10.3
Use of Mosquito Repellant on the Body	56	57.7
Wear Clothes that Cover the Body	32	33.0
Use Condom/Have my Partner Use a Condom	5	5.2
Clean/Scrub Water Storage Containers or Containers with Standing Water	18	18.6
Remove Standing Water	32	33.0
Fumigate my Home	14	14.4
Put Screens on Windows or Doors	9	9.3
